# Multiple esophageal ulcers in a pediatric case of granulomatosis with polyangiitis: A case report

**DOI:** 10.1002/deo2.70089

**Published:** 2025-02-26

**Authors:** Yuki Kimura, Takashi Ishige, Takuya Nishizawa, Yoshiko Igarashi, Yoshihito Saito, Ryusuke Yagi, Maiko Tatsuki, Reiko Hatori, Hayato Ikota, Takumi Takizawa

**Affiliations:** ^1^ Department of Pediatrics Gunma University Graduate School of Medicine Gunma Japan; ^2^ Department of Diagnostic Pathology Gunma University Hospital Gunma Japan

**Keywords:** colonoscopy, esophagogastroduodenoscopy, inflammatory bowel disease, steroid, ulcerative colitis

## Abstract

A 14‐year‐old girl presented with diarrhea and bloody stools was initially diagnosed with infectious colitis and anal fissure. The patient was treated with antibiotics; however, the symptoms persisted and purpura appeared on the patient's lower abdomen. Abdominal computed tomography indicated diffuse wall thickening of the entire colon. A colonoscopy revealed extensive edema, several ulcers, and mucosal friability, resulting in the diagnosis of ulcerative colitis. Blood tests revealed hypoalbuminemia, increased inflammatory marker levels, and high proteinase3 anti‐neutrophil cytoplasmic antibody (PR3‐ANCA) levels. Urinalysis showed hematuria and casts, raising the suspicion of concurrent vasculitis syndrome. Esophagogastroduodenoscopy revealed multiple punched‐out ulcers in the esophagus. Granulomatosis with polyangiitis with gastrointestinal involvement was diagnosed combined with the positive PR3‐ANCA results and skin and renal involvement. Steroid therapy was initiated, leading to the rapid improvement of diarrhea, purpura, and esophageal ulcers. While high PR3‐ANCA levels are occasionally observed in ulcerative colitis, esophageal ulcers in patients with granulomatosis with polyangiitis often result in poor symptoms. Thus, esophagogastroduodenoscopy should be considered in patients with high PR3‐ANCA levels, even in the absence of upper gastrointestinal symptoms.

## INTRODUCTION

Systemic vasculitis, such as granulomatosis with polyangiitis (GPA), can sometimes be linked to gastrointestinal complications, including inflammatory bowel disease. Gastrointestinal lesions occur in 10%−24% of GPA cases and may involve ulcerative colitis (UC), small bowel perforation, and gastrointestinal obstruction.^1^ In children, diagnosing esophageal ulcers can be challenging due to difficulties in symptom expression, increasing the risk of delayed diagnosis. Herein, we report a pediatric case of GPA presenting with UC, purpura, and esophageal ulcers detected via upper gastrointestinal endoscopy.

## CASE REPORT

A 14‐year‐old girl presented with more than 10 bloody diarrhea episodes per day without abdominal pain. On the first day of the illness, the patient experienced diarrhea approximately once per hour, even waking them up at night. On the fourth day of illness, the patient developed a temporary fever, which rapidly subsided. The patient was diagnosed with infectious colitis and anal fissure on the fifth day when they visited a general practitioner and were prescribed antibiotics. However, the symptoms did not improve. On the eleventh day, the patient developed purpura in their lower abdomen. Around this time, the patient had lost 3 kg over a month, and abdominal computed tomography revealed thickening of the entire colonic wall, raising the suspicion of UC. The patient was subsequently referred to our hospital. Following a medical interview on admission, the perinatal history showed that the patient was born at 36 weeks and 6 days of gestation via spontaneous delivery, with a birth weight of 1562 g (−3.0 SD) and a height of 39.8 cm (−2.8 SD). No significant family nor allergy history was noted. The patient's medical history included an episode of bloody stools at 13 years of age and a diagnosis of an anal fissure that immediately improved.

**FIGURE 1:**
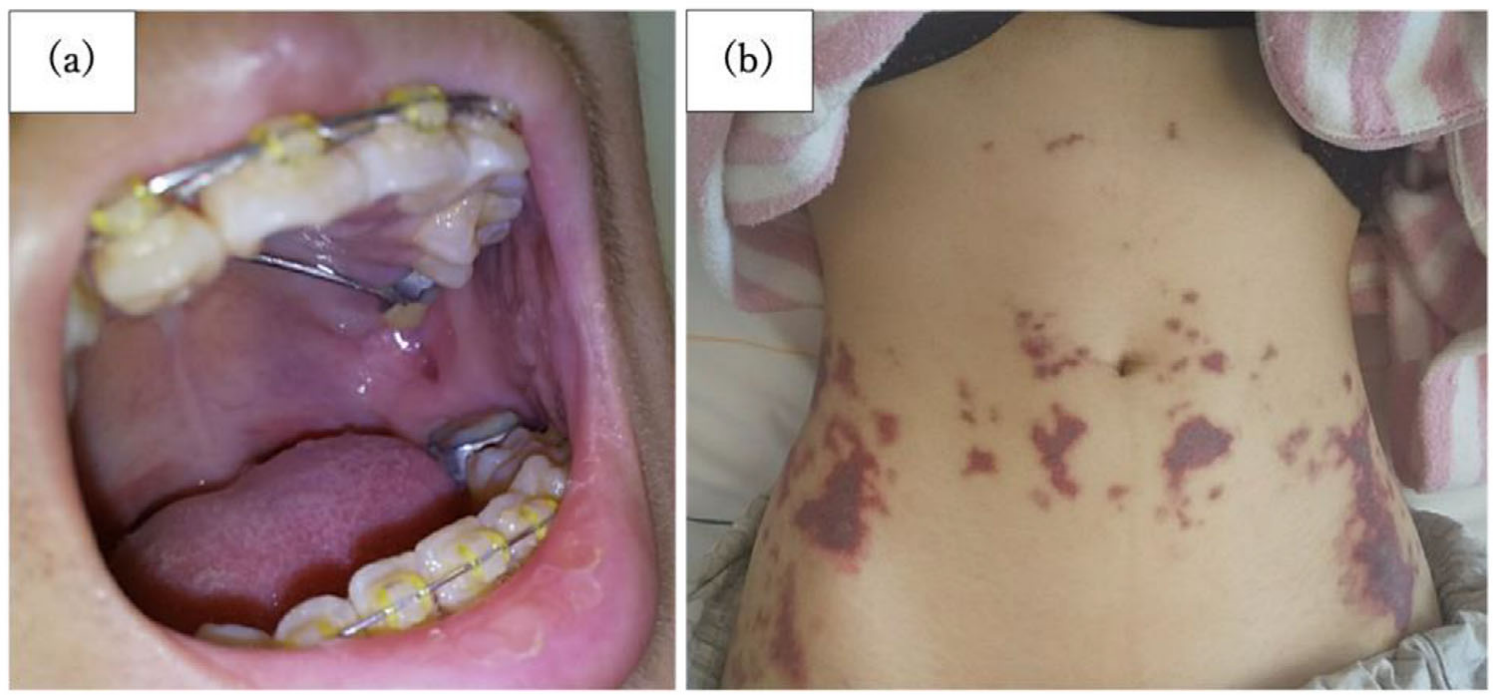
Physical findings on admission: oral ulcers (a) and multiple abdominal purpura (b).

On physical examination, the patient had a height of 150.9 cm, a weight of 36.4 kg, and a heart rate of 103 beats per minute, indicating tachycardia without fever. The patient appeared pale, and anemia of the palpebral conjunctiva was observed. An intraoral ulcer was noted at the site of contact with the orthodontic appliance (Figure 1a). Regarding skin symptoms, multiple purpura were present across the entire abdomen; however, none were observed on the lower extremities or buttocks (Figure 1b).

**FIGURE 2:**
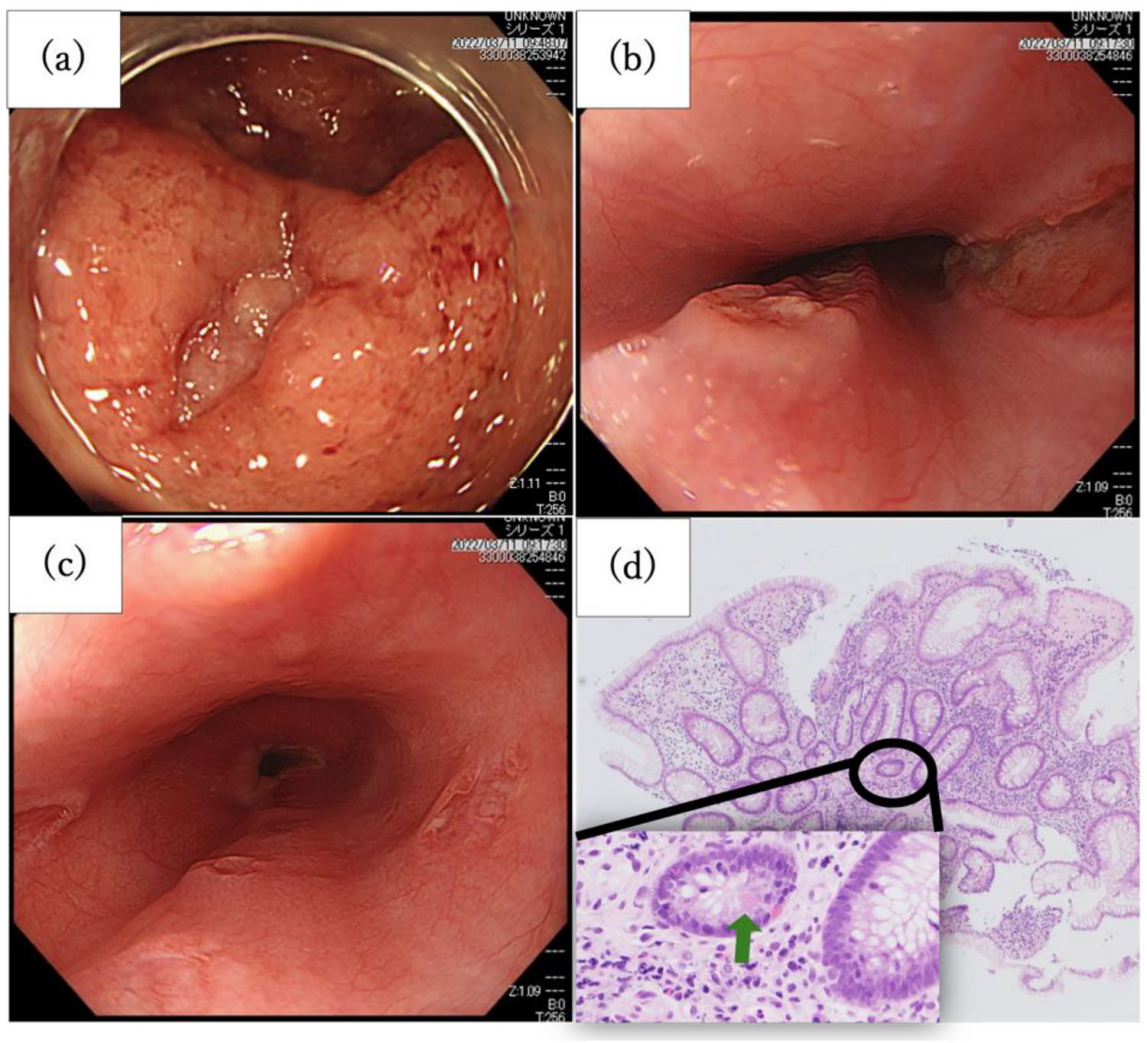
First endoscopic study of the esophagus and rectum (a–c), and the histological findings of the specimens of colonic biopsy (d). Lower gastrointestinal endoscopy showed several similarly shaped deep‐bleeding ulcers in the rectum (a). Upper gastrointestinal endoscopy revealed ulcers with marginal ridges and punching‐like ulcers in the middle (b) of the lower esophagus (c). Hematoxylin and eosin staining of the specimens of the sigmoid colon biopsy. This revealed torsion of the crypts, goblet cell depletion, and Paneth cell metaplasia (arrowhead) (d).

Blood tests revealed increased white blood cells at 15,400 /µL, platelets at 492,000 /µL, and C‐reactive protein (CRP) at 5.37 mg/dL, indicating inflammation. Hemoglobin was mildly low at 11.6 g/dL, and the activated partial thromboplastin time was low at 25.2 s. fibrin degradation products level was elevated and factor XIII activity was reduced to 48%. Low albumin levels were noted at 2.4 g/dL (Table 1). A colonoscopy revealed a diffuse loss of vascular visibility and substantial edema throughout the colon, especially in the hepatic flexure of the transverse colon. Small irregularly shaped ulcers were scattered around the ascending colon and splenic flexure. The mucosa extending from the splenic flexure to the sigmoid colon appeared dark purple and resembled ischemic colitis, suggesting a combination of inflammation and ischemia. Several annular deep ulcers were present from the upper to lower rectum (Figure 2a); however, friability was not evident. No obvious findings were observed in the terminal ileum. Pathological examination of the rectum showed basal plasmacytosis, the diagnosis of UC was confirmed according to the Revised Porto criteria for the diagnosis of pediatric inflammatory bowel disease. Contrastingly, an upper gastrointestinal endoscopy, performed to differentiate between Crohn's disease and other diseases, demonstrated multiple ulcers in the mid‐to‐lower esophagus. Furthermore, punched‐out ulcers that appeared to bulge out were observed in the middle esophagus (Figure 2b,c). Pathological examination of the middle esophagus revealed inflammatory cell infiltration, including neutrophils, around the blood vessels; however, no evidence of vasculitis or granuloma formation was observed (Figure 2d). No ulcerations were observed in the stomach.

**TABLE 1:** Blood test findings on admission.

**WBC**	15,400 /µL	TP	6.1 g/dL	Ferritin	39.2 ng/mL
**Neutrophil**	83.0%	Alb	2.4 g/dL	IgG	1553 mg/dL
**Eosinophil**	1.0%	T‐Bil	0.34 mg/dL	IgA	198 mg/dL
**Monocyte**	4.0%	AST	10 U/L	IgM	70 mg/dL
**Lymphocyte**	12.0%	ALT	10 U/L	CRP	5.37 mg/dL
**Hemoglobin**	11.6 g/dL	LDH	165 U/L		
**Platelet**	49.2 × 10^4^ /µL	ALP	267 U/L	EBV VCA‐IgM	–
**PT‐INR**	1.22	BUN	8.0 mg/dL	CMV‐IgM	–
**APTT**	25.2 sec	Cre	0.42 mg/dL	Fecal occult blood	+
**Fib**	308 mg/dL	Na	137.1 mEq/L	Fecal Calprotectin	8550 mg/kg
**FDP**	12.9 mg/L	K	3.78 mEq/L	MPO‐ANCA	<1.0 mg/dL
**Factor XIII**	48%	Cl	102.1 mEq/L	PR3‐ANCA	217.9 mg/dL

Abbreviations: APTT, activated partial thromboplastin time; CMV‐IgM, cytomegalovirus‐IgM; EBV VCA‐IgM, Epstein‐Barr virus‐virus capsid antigen‐IgM; FDP, fibrin degradation products; Fib, fibrinogen; MPO‐ANCA, myeloperoxidase anti‐neutrophil cytoplasmic antibody; PR3‐ANCA, proteinase3 anti‐neutrophil cytoplasmic antibody; PT‐INR, prothrombin time‐international normalized ratio.

As the pediatric UC activity index score was 75, indicating a severe UC, steroid therapy (prednisolone; 60 mg/day) was initiated immediately. Additional immunological tests revealed a significantly increased proteinase3 anti‐neutrophil cytoplasmic antibody (PR3‐ANCA) level (217.9 mg/dL). The patient was also diagnosed with GPA under the 2022 American College of Rheumatology/ European Alliance of Association for Rheumatology (ACR/EULAR) criteria, given the markedly elevated PR3‐ANCA levels.^2^ Urinalysis revealed a red blood cell count of 400/HPF in the sediment. Additionally, mild proteinuria was observed with a urine protein/creatinine ratio of 0.17. A skin biopsy of the purpura under steroid treatment revealed the formation of microthrombi in the dermis and mild inflammatory cell infiltration, predominantly by lymphocytes. However, fibrinoid necrosis or granuloma formation in the vessel walls was unclear, making it challenging to confirm GPA based on skin pathology. Capsule endoscopy performed on day 21 revealed no obvious lesions in the small intestine.

**FIGURE 3:**
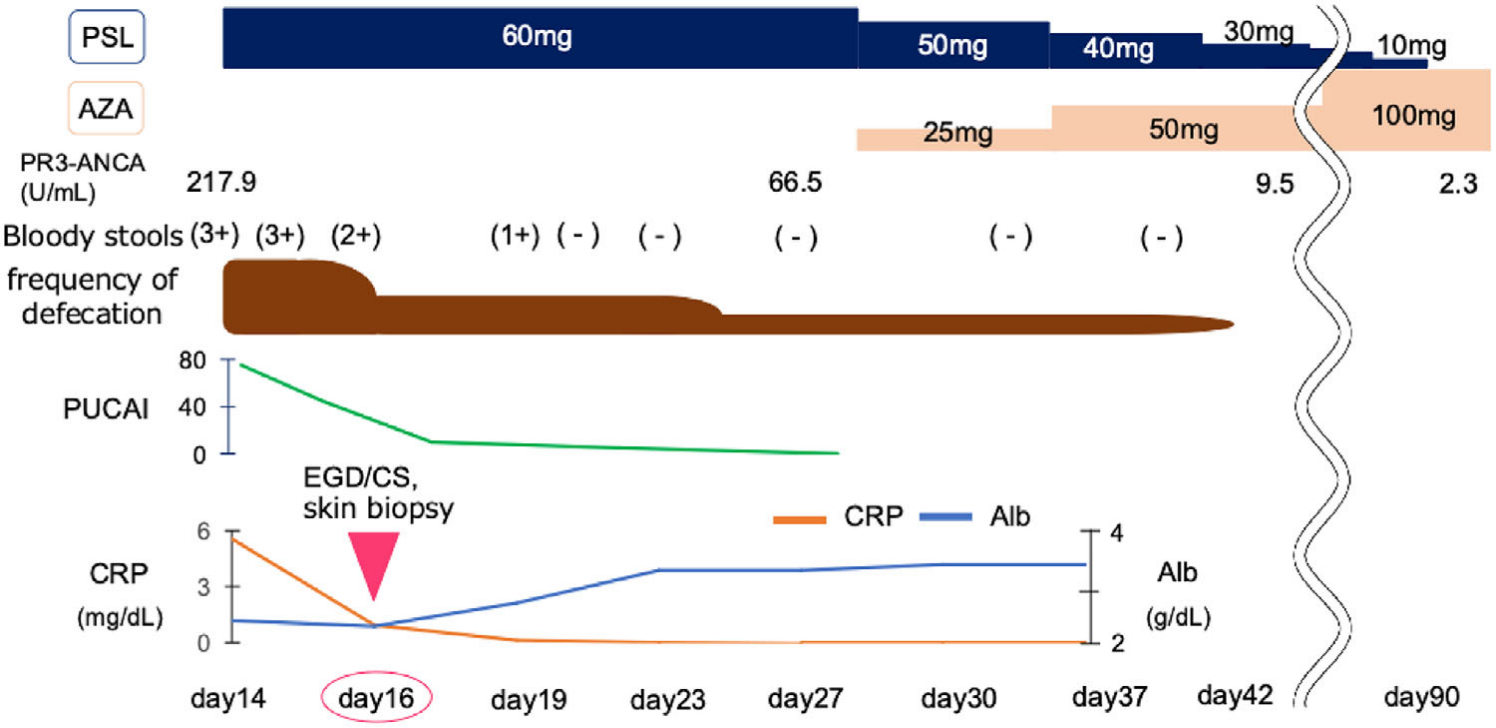
Summary of the main aspects of the reported case. PSL and AZA doses used for treatment are indicated. Trends of proteinase3 anti‐neutrophil cytoplasmic antibody (PR3‐ANCA), bloody stools, defecation frequency, PUCAI, CRP, and Alb are summarized in chronological order. PSL, prednisolone; AZA, azathioprine; PUCAI, pediatric ulcerative colitis activity index; Alb, albumin; CRP, C reactive protein.

After steroid therapy, the symptoms of UC, urine, and skin manifestations, and C‐reactive protein and PR3‐ANCA levels immediately improved (Figure 3). Subsequently, the patient was maintained on azathioprine, and no recurrence of UC or vasculitis existed. A follow‐up esophagogastroduodenoscopy conducted 16 months later revealed complete improvement.

## DISCUSSION

Systemic diseases that cause esophageal ulcers include infectious diseases and collagen vascular diseases. Particularly, cytomegalovirus, herpes virus, candida, tuberculosis, Behçet's disease, and Crohn's disease are well‐known, with Behçet's disease being especially common among collagen diseases.^3^

GPA is an idiopathic primary vasculitis characterized by granulomatous necrotizing inflammation of the small arteries and veins. GPA is rare in pediatrics, but has been reported at around 10 years of age; it is the most common ANCA‐associated vasculitis (AAV) of childhood and up to 3.3% of cases of GPA have an onset prior to the age of 20 years.^4^ According to the 2022 ACR/EULAR Classification criteria for GPA, this case scored 8 points due to PR3‐ANCA positivity and oral ulceration, classifying it as GPA, as a score of ≥ 5 is required.^2^

GPA infrequently affects the gastrointestinal tract compared to other small‐ or medium‐vessel vasculitis reported in the literature, with reports indicating that it occurs in 10%−24% of adults and 12%−42% of children.^1^, ^4^, ^5^, ^6^, ^7^ Gastrointestinal lesions commonly include ulcers, submucosal edema, and ischemic enteritis. The colonoscopy findings in this case partially resembled ischemic colitis, which further supports GPA diagnosis.^8^ Esophageal perforation can be potentially fatal.^9^ In our literature search for GPA with esophageal ulceration, four other cases, including adults, were reported as either confirmed or suspected. One fatality was reported in all cases. Although symptoms like dysphagia and pharyngeal discomfort may be observed, our patient did not demonstrate those symptoms.^6^ Therefore, esophagogastroduodenoscopy should be performed in cases of increased PR3‐ANCA levels, even in the absence of upper gastrointestinal symptoms, to identify asymptomatic esophageal or gastric complications. In other words, physicians should be aware of GPA as a potential cause of esophageal ulcers to prevent delays in diagnosis and treatment.

In adults, GPA‐related esophageal ulcers have been reported, and we believe that GPA contributed more to the esophageal ulcer than UC in this case. However, distinguishing between the two is challenging, as GPA may have influenced the development of UC. Further investigation is warranted to clarify this relationship.

Recently, αvβ6 integrin was reported to be a useful biomarker for the diagnosis of UC.^10^ Although it was not measured in this case, it is expected that the usefulness of this biomarker with respect to GPA‐associated UC may be demonstrated in future studies.

In conclusion, we encountered a pediatric case with esophageal involvement while meeting the diagnosis of GPA according to the ACR/EULAR 2022 criteria and endoscopic and pathologic findings consistent with UC, which have not been reported previously. In cases of unexplained enteritis or UC with increased PR3‐ANCA levels, examining the entire gastrointestinal tract, not just the colon, is crucial through procedures like upper gastrointestinal endoscopy, regardless of the presence of symptoms, to improve prognosis.

## CONFLICT OF INTEREST STATEMENT

The authors declare no conflicts of interest.

## PATIENT CONSENT STATEMENT

The written consent of the patient has been obtained from the parent.

## CLINICAL TRIAL REGISTRATION

N/A.
